# Does Myalgic Encephalomyelitis/Chronic Fatigue Syndrome (ME/CFS) Represent a Poly-Herpesvirus Post-Virus Infectious Disease?

**DOI:** 10.3390/v17121624

**Published:** 2025-12-16

**Authors:** Maria Eugenia Ariza, Irene Mena Palomo, Marshall V. Williams

**Affiliations:** 1 Institute of Brain, Behavior and Immunology (IBBI), The Ohio State University Wexner Center, Columbus, OH 43210, USA; 2 Department of Cancer Biology and Genetics (CBG), The Ohio State University Wexner Center, Columbus, OH 43210, USA; 3 Biomedical Sciences Graduate Program, The Ohio State University College of Medicine, Columbus, OH 43210, USA

**Keywords:** myalgic encephalomyelitis/chronic fatigue syndrome (ME/CFS), deoxyuridine triphosphate nucleotidohydrolase (dUTPase), Epstein–Barr virus (EBV), human herpesvirus 6A (HHV-6A), varicella-zoster virus (VZV), Gulf War Illness (GWI), post-acute sequelae of COVID-19 (PASC)

## Abstract

Myalgic encephalomyelitis/chronic fatigue syndrome (ME/CFS) is a debilitating multisystem illness with unknown etiology. An estimated 17–24 million people representing approximately 1% of the population are afflicted worldwide. In over half of cases, ME/CFS onset is associated with acute “flu-like” symptoms, suggesting a role for viruses. However, no single virus has been identified as the only etiological agent. This may reflect the approach employed or more strongly the central dogma associated with herpesviruses replication, which states that a herpesvirus exists in two states, either lytic or latent. The purpose of this review is to address the role that abortive lytic replication may have in the pathogenesis of ME/CFS and other post-acute viral infections and also to raise awareness that these syndromes might be poly-herpesviruses mediated diseases.

## 1. Introduction

Myalgic encephalomyelitis/chronic fatigue syndrome (ME/CFS) is a debilitating, multisystem illness characterized by post-exertional malaise (PEM), immune dysfunction, muscle pain and weakness, metabolic defects, cognitive impairment, sleep disturbances, and orthostatic intolerance [1,2,3,4,5]. Prior to the COVID pandemic, ME/CFS was estimated to affect 17–24 million people worldwide, representing approximately 1% of the population [6]. However, dismissal and limited awareness of the disease among some general practitioners coupled with inconsistent diagnostic criteria suggest that the true number may be far higher [7,8,9,10,11,12,13,14].

For decades, clinicians and researchers have developed separate case definitions and diagnostic criteria for ME [15,16,17,18,19,20,21,22]. Over this time period, four major sets of clinical case criteria have been established for the diagnosis of ME/CFS: the Fukuda criteria in 1994 [21] with and without the Reeves modification [22], the Canadian Consensus criteria (CCC) in 2003 [17], the International Consensus criteria (ICC) in 2011 [23], and the IOM criteria in 2015 [7], which is the current diagnostic criteria used by the CDC. In 2015, the IOM proposed that the Canadian Consensus criteria be employed for research purposes [7], but the Fukuda criteria has been employed in most studies [24] in spite of criticisms that this case definition is overtly inclusive [25,26]. Jason et al. [26] indicated that those subjects meeting the research criteria were significantly more impaired on a wide variety of symptoms and functional areas when compared to those meeting the clinical criteria. There are no specific biomarkers available to confirm a diagnosis of ME/CFS. Instead, patients are diagnosed by ruling out other illnesses or conditions that could explain their symptoms and by determining whether those symptoms meet established case criteria. At present, there are no approved treatments for ME/CFS.

Research into ME/CFS began long before formal case definitions were established. Early studies date back to the 1930s, when the outbreak of a polio-like illness known as benign myalgic encephalomyelitis was reported [27]. Because of the epidemic nature of the studies, clinicians and researchers suspected a viral cause. Prior to 1980, much of the focus was on persistent enterovirus infections, a hypothesis that remains under consideration as potential etiological agents of ME/CFS today [28,29]. Later, following the Nevada and New York outbreaks, the attention shifted to a human herpesvirus, Epstein–Barr virus (EBV), as patients exhibited symptoms resembling a chronic mononucleosis-like syndrome. These investigations centered on individuals with prolonged fatigue, and the term “post-fatigue syndrome” was coined. Reports indicated that more than 70% of chronic fatigue cases occurred following an “infectious, flu-like illness” [30,31,32], reinforcing the premise that a single virus might be the cause of ME/CFS.

## 2. Are the Symptoms of a Subset of Patients with ME/CSF the Result of a Post-Virus Infection?

Several viruses including the coronaviruses (SARS, SAR-C0V-2), influenza virus, Ebola virus, Dengue virus, enteroviruses (polio and Coxsackie B), Chikungunya virus, West Nile virus, Ross fever virus, and several herpesviruses (EBV, human herpesvirus 6 (HHV-6), human herpesvirus 7 (HHV-7), and varicella-zoster virus (VZV)) have been associated with post-acute infection syndromes (PAISs) [31]. While severity is variable, patients with PAIS exhibit flu-like symptoms of exertion intolerance, disproportionate levels of fatigue, neurocognitive and sensory impairment, unrefreshing sleep, and myalgia/arthralgia [31]. In the case of ME/CFS, the viruses most closely associated with this syndrome have been enteroviruses and several human herpesviruses. Enterovirus, a genus within the family Picornaviridae, comprises enteroviruses, coxsackieviruses, rhinoviruses, polioviruses, and echoviruses. These viruses cause a wide variety of illnesses ranging from the common cold to poliomyelitis and aseptic meningitis. In humans, they are among the most common infectious agents worldwide. More than 90% of primary infections are asymptomatic or result in a mild febrile illness. Primary infections usually occur by 5 years of age, and over 90% of the cases occur in children younger than 14 years. Enteroviruses produce acute cytolytic infections generally cleared by the host adaptive immune response. While somewhat controversial [33,34], it has been proposed that the development of persistence of non-cytolytic variants may occur following acute infections with enterovirus B serotypes (coxsackievirus and echovirus) [35,36,37]. Prior to 2010, there were numerous studies investigating the possible relationship between enterovirus infection and ME/CFS [38]. Since 2010, the number of studies has subsided, with only four studies, two of which were reviews [28,29,39,40], suggesting the interest might be fading.

Conversely, in the past decade or so, there has been an increased research interest regarding the potential role(s) of some herpesviruses, particularly EBV and HHV-6A/B, in ME/CFS. Numerous studies, both historical and ongoing, reflect this heightened focus, underscoring the persistent effort to clarify their role in the syndrome’s development.

There are nine members of the *Herpesviridae* that infect humans, and they are classified into three subfamilies: the α-herpesviruses (herpes simplex types 1 and 2 [HSV-1 and 2] and VZV], the β-herpesviruses (human cytomegalovirus [HCMV], HHV-6A and 6B and human herpesvirus-7 [HHV-7]), and the γ-herpesviruses (EBV and human herpesvirus-8 [HHV-8]) (Table 1). While individual herpesviruses can be distinguished based on cellular tropism, serology, and genomic DNA sequence, a common feature shared by members of the *Herpesviridae* family is the ability to establish life-long persistent infections. For most herpesviruses, primary infection occurs in childhood and is typically asymptomatic, with prevalence increasing with age. More than 90% of the adult population is persistently infected with EBV, HHV-6A/B, HHV-7, and VZV. Notably, these viruses are associated with a wide range of diseases in both immune-competent and immune-suppressed individuals, posing a greater burden than once recognized. Numerous studies have explored EBV and HHV-6 as possible triggers of ME/CFS, but conflicting findings regarding their presence in patients led Soto and Straus [41] to conclude that evidence for herpesviruses involvement in ME/CFS was fading. More recent research, however, suggests otherwise, as discussed in this manuscript.

## 3. If ME/CFS Is Triggered by a Virus in Some Cases, Why Has It Not Been Identified? Factors to Consider

Research investigating the viral etiology of ME/CFS has generally been epidemiological in nature, relying on serological evidence, increased viral load, or both to suggest causation. Most of these studies sought to identify a single etiological agent, yet none has been definitively identified/established. Several factors contributed to this problem: Variations in the case criteria used for diagnosis have resulted in extreme heterogeneity in the study populations, the cohorts’ size is often too small for robust statistical analysis, and the different endpoints measured. Perhaps more importantly are the experimental approaches and clinical samples employed. Most studies have focused on detecting antibodies, typically IgG, against specific viral proteins (antigens), or on viral load assessment using plasma or PBMCs. Each approach has inherent limitations, complicating efforts to reach firm conclusions.

### 3.1. Lack of Standardized Criteria to Designate Individuals as Healthy Controls

Most studies define healthy control populations as individuals lacking evidence of the disease under investigation. Yet, the virome of healthy controls is not characterized prior to their inclusion in a study, which is a critical oversight when studying virus-associated illnesses. Without such screening, biases may be introduced that further complicate interpretations of results. The human virome is a highly complex ecosystem, unique to each person, and shaped by factors such as age, diet, gender, and interactions with other members of the microbiome [49,50]. Despite its importance, research into the virome and its interplay with the broader microbiome is very limited. Metagenomic analyses have revealed that herpesviruses are commonly present in healthy individuals across multiple body compartments, including blood, gastrointestinal tract, lungs, oral cavity, nervous system, reproductive organs, skin, and urinary tract, while picornaviruses have been found primarily in the gastrointestinal system [49,50,51]. However, there is no evidence that control groups included in ME/CFS studies are screened for persistent infections which could influence baseline values. Consistent with this, RNA sequencing (RNA-seq) and metagenomic studies of ME/CFS patients’ feces, blood, and saliva have not identified significant differences between their virome and those of healthy controls [51,52,53,54,55,56,57]. Given the high prevalence of some herpesvirus infections (>90% for EBV, HHV-6, HHV-7, and VZV) in the adult population, it is not surprising that RNA-seq and metagenomic analyses fail to show statistically significant differences between healthy controls and ME/CFS cases. This aligns with two recent comprehensive studies reporting that ME/CFS cases and healthy controls exhibit similar exposure to most common pathogens [58,59].

### 3.2. Serological Studies

In general, most studies investigating the potential role of enteroviruses and herpesviruses as triggers for ME/CFS have relied on commercially available ELISAs or ELISA-like platforms to detect antibodies, typically IgG, against specific virus proteins. In some cases, these assays use highly purified viral antigens such as VP1 (a capsid protein of enteroviruses) or viral capsid antigen (VCA) of EBV. In other cases, however, they employ relative crude preparations (“purified viral lysates”). Because the viral lysates contain a mixture of many viral antigens, these ELISAs cannot determine nor distinguish the host’s immune response to any single virus protein. This is important since the antigenic properties of virus proteins are not the same. As demonstrated by Vaider-Shalt et al. [60], over the course of their evolution, HSV-1, EBV, and human cytomegalovirus (HCMV) have decreased the number of epitopes present in virus proteins to help them avoid immune detection. Thus, the ability of a virus protein to generate an antibody response is dependent on the amount of protein present in the host and its antigenicity. Not surprisingly, serological studies employing viral lysates have led to conflicting results. Recent studies using advanced methodologies such as peptide microarray [61] and suspension multiplex immunoassay [62] have shown that the EBV-specific IgG response in ME/CFS patients did not differ significantly from that of healthy controls. These findings are consistent with numerous reports highlighting that the interpretation of EBV serological data [63] and, by extension, serological data for other herpesviruses and potentially enteroviruses [64] is inherently complex and requires caution. Although enterovirus-specific IgM and IgG assays have been developed, their practical utility is limited due to antigenic cross-reactivity among different serotypes and the high prevalence of enteroviruses in the general population [64,65,66,67,68,69,70,71].

### 3.3. Use of Viral Load as a Requirement to Demonstrate a Viral Infection

A range of platforms has been employed to directly determine viral genome levels in ME/CFS patients and healthy controls. Although no studies to date have specifically examined enterovirus load in ME/CFS patients, several studies have consistently reported no increase in EBV and/or HHV-6 viral load among affected ME/CFS patients [72,73,74,75]. This raises an important question: do these findings reflect limitations in methodologies/approaches, or are they shaped by the prevailing dogma/assumptions about how these viruses replicate and persist in vivo?

### 3.4. Abortive Lytic Replication (ALR)

A central concept regarding the biology of herpesviruses is that two distinct phases of viral gene expression exist, either latency or lytic replication where virus progeny are produced. However, there is accumulating data to suggest that in vivo a third state exists in which the virus undergoes abortive lytic replication (ALR). In ALR, the virus initiates the lytic cycle but does not fully complete it, resulting in limited viral replication and no production of infectious virions. During this process, some immediate-early and early lytic genes are expressed, though the specific genes expressed vary across herpesviruses. Although early studies showed ALR in EBV [76,77,78,79,80,81,82], subsequent studies have shown that ALR also occurs in cells infected with HCMV [83,84], HHV-6 [85], HHV-8 [86,87,88], HSV-1 [89,90,91,92], and VZV [93,94,95]. An essential aspect of ALR is that it does not lead to an increase in virus load [76,78,80,81,83,90,96,97]. This characteristic of ALR provides a clear rationale for the lack of significant differences in viral load between individuals with ME/CFS cases and healthy controls. Despite evidence of lytic gene activity, the replication process aborts before producing infectious virions, thus preventing measurable changes in overall viral burden [76,77,78,79,80,81,82,83,84,85,86,87,88,89,90,91,92,93,94,95,96,97].

More importantly, although these immediate-early and early genes regulate virus replication, these same proteins also modulate host genes involved in immune evasion, cell proliferation, the establishment and maintenance of latency, and apoptosis. Elevated antibodies to the deoxyuridine triphosphate nucleotidohydrolases (dUTPase) encoded by the early genes of EBV (BLLF3), HHV-6 (U45), and VZV (ORF8) were reported in both longitudinal and single serum samples from patients with ME/CFS compared to controls [96,97], suggesting that ALR may be occurring in these patients. Moreover, it was recently reported that the EBV dUTPase protein is expressed in postmortem brain tissues of ME/CFS cases but not in non-ME/CFS control tissues [98], highlighting the potential of herpesvirus dUTPases as distinctive biomarkers for ME/CFS.

## 4. Is ME/CFS the Outcome of Synergistic Interactions Between Multiple Herpesviruses?

The premise that ME/CFS may be the result of a poly-herpesvirus infection is not new. Lerner et al. [99,100] proposed that ME/CFS is caused by EBV, HCMV, and HHV-6 in single or multiple virus co-infections. While no research has been specifically designed to directly test this hypothesis, several serological and viral load-based studies have shown evidence of increased reactivation of several herpesviruses in ME/CFS patients. Manian [101] found that among ME/CFS patients (*n* = 20) diagnosed according to the Holmes et al. [18] criteria, 20% exhibited elevated IgG antibodies against EBV, HHV-6, and HSV1/2. However, these findings were not statistically different from age- and gender-matched controls. Similarly, Cameron et al. [72], studying ME/CFS patients (*n* = 10; Age 34 ± 15; 7 females 3) from the Dubbo Infection Outcome Study (post-infectious fatigue), reported that all patients were IgG seropositive for HHV-6, and 50% were seropositive for HCMV, yet these rates did not differ significantly from the control group. In contrast, a larger study by Chapenko et al. [102] which assessed both serological and viral load found that 26.85% of ME/CFS patients (*n* = 108) diagnosed using the Fukuda case definition [21] exhibited active co-infections with HHV-6, HHV-7, or parvovirus B19, whereas none of the controls (*n* = 90) showed evidence of such active co-infections. Similar findings were reported using a larger cohort of ME/CFS patients (*n* = 200) diagnosed using the Fukuda case definition [21]. In these studies, latent infection or co-infection with HHV-6A/B, HHV-7, and parvovirus B19V was observed in 51.5% of patients and in 76.7% of healthy control individuals (*n* = 150). In contrast, active infection was observed in approximately 45% of ME/CFS patients compared to only 8.7% of healthy controls [103]. Likewise, Briese et al. [56], using a metagenomics study of saliva from ME/CFS patients (*n* = 106), found that 30% of cases were coinfected with two herpesviruses and 21% with at least three. Similar results were observed in the control group (*n* = 91; 34 and 26%, respectively). Halpin et al. [96] analyzed both single serum samples (*n* = 55) and longitudinal samples (*n* = 74, 4 samples each) from ME/CFS patients diagnosed using the Fukuda with Reeves modification as well as the Canadian case definitions. Their findings revealed that patients could be stratified into two distinct subgroups: those exhibiting IgG antibodies to the herpesviruses encoded dUTPase and those who did not. Furthermore, the study showed that a significant percentage of ME/CFS patients (30.91–52.7%) were simultaneously producing IgG antibodies against multiple human herpesviruses (EBV, HHV-6, and VZV) dUTPases compared to controls (*n* = 151; 17.21%) [96]. In a follow-up study involving a different patient cohort, the same group found evidence of significant reactivation of multiple herpesviruses (EBV, HHV-6, and VZV) in 50% of ME/CFS female cases (*n* = 40; 873 longitudinal samples over 46–80 days), based on elevated dUTPase IgG antibodies. In contrast, only 6% of the control cohort (*n* = 16) showed reactivation [97]. More importantly, anti-dUTPase antibody levels directly correlated with fatigue and pain, two hallmark symptoms of ME/CFS. Finally, there have been several additional reports of simultaneous reactivation of herpesviruses (EBV, HSV-1, HHV-6, and VZV) in ME/CFS patients, but these studies did not provide individual patient data [62,104,105].

Additional support for the hypothesis that ME/CFS may represent a poly-herpesvirus syndrome comes from several double-blind, placebo-controlled studies demonstrating that long-term therapy (6 months) with the herpesvirus-targeting antivirals valaciclovir or valganciclovir led to improved clinical symptoms and reduced serum antibody titers among ME/CFS patients infected with EBV or with combinations of EBV, HCMV, and HHV-6 [98,106,107,108,109,110,111].

Interestingly, adolescents who develop heterophile antibody-positive infectious mononucleosis (IM) caused by EBV [112] have a 12–23% probability of developing severe ME/CFS if they meet >1 set of criteria (Fukuda, Canadian, and Institute of Medicine) for ME/CFS [113,114,115,116,117,118]. Serological studies have also reported that, in addition to EBV, these patients may also be co-infected with HCMV, HSV-1, and/or HHV-6 [119,120,121,122,123]. Bertram et al. [120] reported that 39.5% of the serum samples from 215 patients with infectious mononucleosis showed active co-infections of EBV and HHV-6. Furthermore, Al Tabaa et al. demonstrated that ALR is a key feature of EBV in patients diagnosed with infectious mononucleosis [78]. However, the potential contribution of these viral interactions to the pathogenesis of ME/CFS remains unclear and requires further investigation.

Interactions between viruses and members of the microbiome in vivo is an understudied area; however, evidence suggests that such interactions can be beneficial, neutral, or detrimental, thereby influencing the clinical course of disease [124,125,126,127,128,129,130,131]. Similarly, concurrent reactivation of multiple herpesviruses in patients is considered a rare event and has only been reported in cases where clinical symptoms are present. The best example of a syndrome caused by the sequential reactivation of multiple herpesviruses is Drug Induced Hypersensitivity Syndrome (DIHS)/Drug Reaction with Eosinophilia and Systemic Symptoms (DRESS). DIHS/DRESS is a rare multiorgan, systemic, severe hypersensitivity reaction to a drug that can affect the skin, blood, and any internal organs, most commonly the liver, kidneys, lungs, and heart [128,129]. The cause of DISH/DRESS, which occurs in immunocompetent individuals, is multifactorial, involving drug-exposure, genetic predisposition, viral reactivation, and the immune system response. The sequential reactivation of HHV-6 followed by EBV and HCMV is a hallmark feature implicated in the etiology of DIHS/DRESS. While EBV reactivation can occur initially in some cases, HHV-6 is most often the initial herpesvirus reactivated [129,132]. Systemic complications occur in 15–25% of patients and are classified into two categories: infectious and autoimmune. Among the infectious complications, HCMV-induced pneumonia is associated with poor outcomes. In contrast, reactivation of HHV-6 and EBV has been linked to the development of autoimmune disease [133].

There are multiple reports in the literature documenting concurrent reactivation of multiple herpesviruses in vitro and in vivo [96,97,134,135,136,137]. With the exception of DIHS/DRESS patients, it remains unclear whether multiple herpesviruses reactivate in vivo simultaneously or in sequence. Concurrent reactivation of multiple herpesviruses in vivo may be the result of immunosuppression driven by an expansion of regulatory CD4^+^ T suppressor cells (Tregs) or by CD8^+^ T cell exhaustion. While herpesviruses have been reported to increase Treg levels following infection [138,139,140,141], studies examining ME/CFS patients have reported conflicting results regarding whether Tregs are elevated in this population [142,143,144].

**Figure 1:**
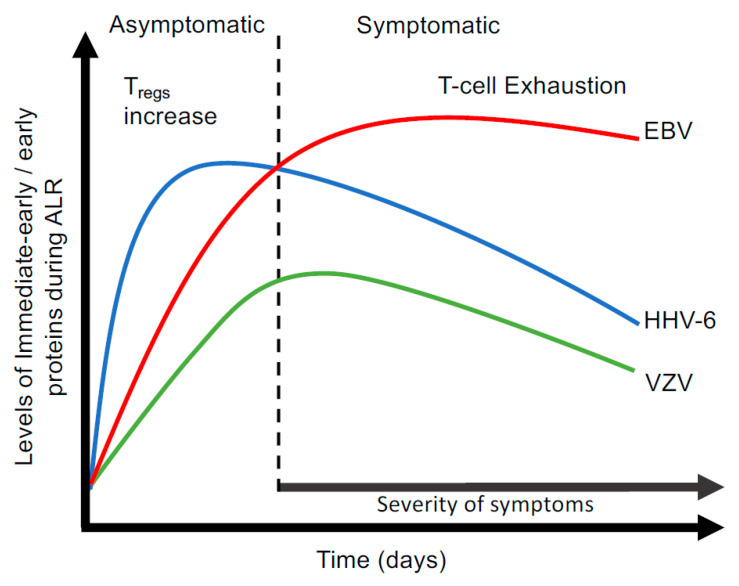
Hypothetical interactions between sequential reactivation of several herpesviruses undergoing abortive lytic activation and the immune system and how these interactions contribute to symptomology observed in a cohort of patients diagnosed with ME/CFS.

Loebel et al. [74] reported an impaired EBV-specific T cell response and diminished IFN-γ production in ME/CFS patients. A recent study conducted in post-infectious-ME/CFS (PI-ME/CFS) [59] and ME/CFS patients revealed CD8^+^ T cell dysfunction [145] which may result in T cell exhaustion [146,147]. Notably, we have previously shown that EBV dUTPase alters T cell function in vitro and in vitro [148,149,150]. These dUTPase effects are consistent with ALR, and we proposed that the ALR of a herpesvirus, likely HHV-6, results in the chronic production of the dUTPase along with other virus proteins. These proteins, through the expression of cross-reactive epitopes, may drive T cell exhaustion [151,152,153], thereby triggering the sequential reactivation of other herpesviruses that contribute to hallmark ME/CFS symptoms, including fatigue, PEM, and cognitive impairment. Furthermore, disease severity is related not only to the number of herpesviruses reactivated but also the amplitude of this reactivation cascade and the duration of CD8^+^ T cell exhaustion (Figure 1). Nonetheless, further studies are needed to substantiate this premise.

## 5. Could Other Multisystem Illnesses Such as Gulf War Illness (GWI) and Post-Acute Sequelae of COVID-19 (PASC) Be Poly-Herpesvirus Infections?

### 5.1. Gulf War Illness (GWI)

Approximately one-third of the US veterans returning from the Operation Desert Storm conflict (8 August 1990–31 July 1991) are afflicted with a chronic multisystem disorder known as Gulf War Illness (GWI) [154,155]. Characterized by severe and debilitating symptoms including fatigue, musculoskeletal pain, and cognitive problems, GWI cases exhibit a significant overlap in symptoms with ME/CFS. Multiple hypotheses regarding the etiology of these symptoms have been proposed, but the etiological agents remain unknown. Studies to examine the potential role of viruses in GWI are limited. Vojdani and Thrasher [156] showed a statistically significant increase in IgM and IgG antibodies against EBV (VCA-IgM); HCMV (IgG); HSV-1 (IgG) HSV-2 (IgG); HHV-6 (IgG); and VZV (IgG) in GWI patients compared to controls. However, individual patient values were not reported. Maloney et al. [157] proposed a latent herpesvirus immune inflammatory response model for chronic multisystem illnesses (CMIs). CMI is a broad term used by the Department of Veterans Affairs to refer to complex conditions of unknown etiology characterized by persistent symptoms that lack a clear medical diagnosis and include veterans diagnosed with GWI and/or ME/CFS. Bast E et al. [158] suggested that GWI might be a post-viral syndrome resulting from pre-exposure to Middle East respiratory syndrome coronavirus (MERS). Halpin et al. [96] showed that a significant percentage of patients with GWI (29.34%) were simultaneously producing antibodies against multiple human herpesviruses-encoded dUTPases compared to controls (17.21%). Notably, GWI patients exhibited significantly higher levels of antibodies to the HHV-6 dUTPase than controls (*p* = 0.0053). Further analyses of longitudinal sera samples from two additional military veteran cohorts diagnosed with CMI or GWI revealed an increased prevalence of IgG (55% and 83%, respectively) and IgM antibodies (80–90% and 100%, respectively) to the dUTPase protein encoded by EBV, HHV-6A, and VZV compared to age- and gender-matched healthy controls (5% and 7% for IgG and IgM, respectively, *p* < 0.001) [159]. These findings indicate the importance of virus dUTPase, expressed as an early gene product during both lytic (productive) and abortive lytic (non-productive) replication of herpesviruses and which has novel immune- and neuro-modulatory functions, as a potential biomarker and key contributor to the pathophysiology of patients with CMI including GWI and ME/CFS [148,160,161,162,163,164,165,166,167,168,169,170,171,172].

### 5.2. Post-Acute Sequelae of COVID-19 (PASC)

Following SARS-CoV-2 infections, a significant number of individuals develop “Post-acute Sequelae of COVID-19 (PASC),” a syndrome characterized by profound fatigue as well as immune and neurocognitive dysfunction that persists for at least 3 months following diagnosis without any alternative explanation for the symptoms. While PASC is initiated by SARS-CoV-2, the underlying mechanisms contributing to the development of PASC remain unclear. Gentilotti et al. [173] identified four clinical phenotypes of PASC, with the dominant one resembling a chronic fatigue-like syndrome phenotype. PASC patients in this phenotype exhibited symptoms such as PEM, fatigue, and cognitive dysfunction identical to those seen in patients with ME/CFS [174,175,176]. Furthermore, it has been estimated that more than 40% of patients diagnosed with PASC could also meet the clinical criteria for ME/CFS [177,178,179,180]. Although several hypotheses have been proposed to explain the mechanisms underlying PASC, at this time, none have been validated. One hypothesis is that the clinical symptoms in PASC may not be a direct result from SARS-CoV-2 but rather from EBV reactivation triggered by COVID-19-induced inflammation [181]. In support of this hypothesis, numerous studies have reported serological and viral load evidence of reactivation of EBV, and other herpesviruses, during both acute SARS-CoV-2 infection and in PASC patients [182,183,184,185]. Furthermore, Su et al. [186], using a longitudinal multi-omics approach, reported that EBV reactivation was one of four biological factors associated with PASC development. Similarly, Peluso et al. [187] found a strong association between EBV reactivation and the fatigue associated with PASC. Verma et al. [188] further showed that EBV lytic replication induced the expression of ACE2 and enhanced SARS-CoV-2 entry into epithelial cells, suggesting that EBV reactivation may also contribute to COVID-19 and possibly PASC. Several studies have reported a correlation between EBV reactivation and disease severity in both COVID-19 and PASC [182,189,190,191,192,193,194,195,196,197,198]; however, conflicting data have also been reported [98,194]. Interestingly, several studies, including ours, have linked EBV reactivation with a subgroup of ME/CFS patients whose symptoms resemble those shown in some PASC patients [60,71,72,73,96,97,180,181,182,183,184,185,186,187,199,200,201].

## 6. Future Directions

**Figure 2:**
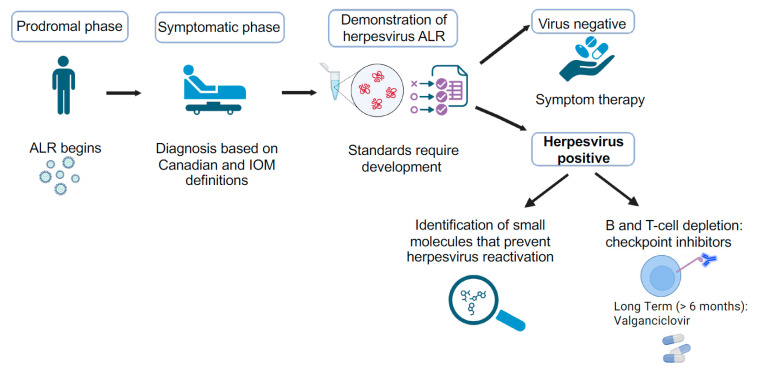
Flow diagram depicting protocols that need to be developed and followed for the accurate diagnosis and treatment of patients with ME/CFS caused by herpesvirus reactivation.

There is sufficient data to demonstrate that ME/CFS patients can be divided into at least two subgroups: those whose illness is not triggered by a virus infection and those whose are. The latter group should be referred to as patients with post-infective ME/CFS (PI-ME/CFS). This classification is necessary because while the symptomology exhibited by patients in these subsets might be similar, the mechanism(s) driving the disease process will be different, and thus, different therapeutic approaches might be required. Putative viruses associated with PI-ME/CFS must be identified not based on viral load but instead on the concepts that abortive lytic replication and co-infections are responsible for this disease. This will require appropriate screening of ME/CFS patients for the presence of abortive-lytic replication. Screening can be performed using either qRT-PCR to quantitate expression of early genes or by using a serological approach to determine the antibody response to multiple proteins encoded by early genes (Figure 2). Early proteins that could be used for this purpose include viral DNA polymerase, viral DNA polymerase accessory protein, and the dUTPase, to name a few. Likewise, individuals classified as “healthy controls” should also be screened, since the expression of early virus proteins would suggest they are asymptomatic carriers and therefore not truly representative of “healthy controls.” To improve research consistency, a definitive ME/CFS case definition for researchers must be established, possibly using a combination of the Canadian and IOM case criteria, to reduce patient population heterogeneity and enhance data reproducibility.

It is essential to pursue more mechanistic and hypothesis-driven research to elucidate the pathophysiological effects of these viruses in PI-ME/CFS. Priority should be given to studies investigating synergistic interactions within the virome, particularly between members of the herpesviruses, as they play a role in the initiation and/or progression of ME/CFS. These investigations face limitations, since appropriate animal models for ME/CFS do not exist and human herpesviruses generally do not naturally infect common laboratory rodents. Consequently, such interactions must be identified and studied directly in patients. Advanced techniques such as bulk RNA sequencing and single-cell RNA sequencing (scRNA-seq) offer powerful tools to explore viral synergies and host–virus interactions. Insights gained from these approaches could improve diagnostic accuracy and guide the development of targeted therapeutics.

It has been reported that there is a positive correlation between the levels of antibodies against the herpesvirus dUTPases and the severity of pain and fatigue in patients with ME/CFS [97]. This raises the question of whether disease severity in these patients may be linked to the number of herpesviruses reactivated—a critical issue that requires further investigation.

It has been suggested that future studies into viral infections in ME/CFS should “focus on adaptive immune responses rather than surveillance for viral gene products” [56]. However, this perspective rests on the assumption that an increase in viral load is necessary to demonstrate viral pathogenesis. This assumption overlooks evidence from advanced technologies showing that many dynamic viral infections are abortive lytic in nature. In such cases, numerous viral proteins are expressed that may contribute to disease pathogenesis even without an increase in viral load.

DNA viruses, including human herpesviruses, encode a wide array of proteins. These proteins have diverse effects on host cells, such as promoting cell proliferation, inhibiting apoptosis, and evading immune responses. Identifying the specific viral gene products responsible for fatigue, pain, cognitive dysfunction, and post-exertional malaise (PEM) will be essential for developing reliable biomarkers and targeted therapies. Continued research in this area is therefore critical.

Finally, if simultaneous abortive lytic replication of multiple herpesviruses is a key driver in certain ME/CFS patients, then targeted treatment protocols could be developed for this PI-ME/CFS subgroup. Such approaches might include combinations of antiviral agents (e.g., ganciclovir), cell-depleting therapies such as rituximab, checkpoint inhibitors, or agents designed to prevent viral reactivation.

## Figures and Tables

**Table 1:** Human herpesviruses.

Virus	Prevalence (%)	Age of Primary Infection	Site of Productive Infection	Site of Latency	Common Disease
HSV-1 [42,43]	70	0.5–5 yrs	Epithelial cells	Trigeminal Ganglia	Oral herpes
HSV-2 [42,43]	20–25	15–49 years	Epithelial cells	Sacral Ganglia	Genital herpes
VZV [42,43]	>95	5–9 years	Epithelial cells	Dorsal Root Ganglia	Chickenpox and zoster
HCMV [42,44]	50–>90	5–40 years	Epithelial and Endothelial cells	Myeloid Cell Linage	Immunosuppressed Transplant
HHV-6A [42,45]	>95	0.5–2 years	CD4^+^ T cells	Monocyte/Macrophage Cell Linage	Immunosuppressed Transplant
HHV-6B [42,45]	>95	0.5–2 years	CD4^+^ T cells	Monocyte/Macrophage Cell Linage	Infantum roseola (sixth disease)
HHV-7 [42,46]	>95	1.5–3 years	CD4^+^ T cells	Monocyte/Macrophage Cell Linage	Neurological ?
EBV [42,47]	>95	3–4 years 10–30 years	Epithelial and Plasma cells	Memory B cell	Infectious mononucleosis, various lymphomas, and gastric carcinoma
HHV-8 [42,48]	5–50	4 years in endemic areas	Epithelial, Epithelial, and Plasma cells	Epithelial, Endothelial, and B cells	Kaposi lymphoma, primary effusion lymphoma, and multicentric Castleman’s Disease

## Data Availability

No new data were created or analyzed in this study. Data sharing is not applicable to this article.
